# Effectiveness of Structured Feedback Training on Student’s Performance in Formative Assessment of Physiology Practicals: A Comparative Study

**DOI:** 10.7759/cureus.80176

**Published:** 2025-03-06

**Authors:** Smita A Shinde, Uttama U Joshi, Nisha C Karanje, Sunita S Ingale, Hemlata B Munjappa, Atish B Pagar

**Affiliations:** 1 Department of Physiology, Bharati Vidyapeeth (Deemed to be University) Medical College and Hospital, Sangli, IND; 2 Department of Anatomy, Bharati Vidyapeeth (Deemed to be University) Medical College and Hospital, Sangli, IND; 3 Department of Microbiology, Bharati Vidyapeeth (Deemed to be University) Medical College and Hospital, Sangli, IND

**Keywords:** assessment in medical education, competency based assessment, competency-based medical education (cbme), formative assessment, formative feedback, physiology, structured feedback

## Abstract

Introduction: Formative assessment (FA) followed by feedback plays a crucial role in the constant learning and acquisition of skills. A lack of awareness of the importance and knowledge of structured feedback can lead to its omission or improper delivery by faculty, making it ineffective. It can affect the performance of students in clinical practice in the future. Moreover, a lack of receptivity and internalization of the feedback by students leads to a gap between students' performance and teachers' expectations. Thus, improving learning necessitates awareness and knowledge of structured formative feedback for both teachers and students.

Aim and objectives: This study aimed to evaluate the effect of training of structured formative feedback on students’ performance during FA of hematology and clinical physiology practicals and to assess the perception of faculty and students towards it.

Methodology: According to Competency-Based Medical Education (CBME) guidelines, in Phase I of the Bachelor of Medicine, Bachelor of Surgery (MBBS) program, FA during physiology practicals is conducted regularly in the form of an objective structured practical examination (OSPE) and an objective structured clinical examination (OSCE) followed by feedback on performance. A focused group discussion was conducted to assess the perception of faculty and students toward structured formative feedback, followed by training for faculty and students about how to deliver and receive structured formative feedback, respectively. Every FA of physiology practicals prompted the delivery of structured one-to-one feedback using defined steps. FA scores before and after training sessions were analyzed to see how training faculty and making students aware of structured feedback affected students’ performance of skills in practicals. We assessed faculty and student perceptions of structured formative feedback after the training session using pre-validated questionnaires.

Results: The results showed that FA scores got significantly better (p-value < 0.001) after training sessions. This means that structured formative feedback led to better skill performance. About 90-100% of students felt that structured feedback given in a stepwise manner by faculty is more effective, solution-oriented, and will help improve their skills. Students expressed satisfaction with the delivery of feedback, citing adequate time, a friendly approach, and meeting their expectations. The majority of faculty agreed that feedback was delivered in a structured manner after training sessions for both positive and negative performance points, enhancing self-assessment and the exchange of ideas between student and teacher.

Conclusion: Training for faculty and sensitization of students leads to the delivery of formative feedback in a structured manner, which helps students to understand and rectify mistakes in the performance of skills. Self-assessment by the students increases acceptance of negative feedback in the right way to overcome shortcomings. A change in the attitude of students toward learning leads to better skill performance and scoring in summative assessments and develops self-learning qualities in them. Thus, structured feedback helps students overcome incompetence and achieve desired competencies in the CBME curriculum.

## Introduction

Learning is a two-way process that can be enhanced by understanding the student's learning needs and modifying the teaching methods accordingly. To create efficient practitioners for a stronger healthcare system, medical education in India shifted from a teacher-centered traditional curriculum to a learner-centered Competency-Based Medical Education (CBME) [1,2]. CBME introduces various modern teaching methods like case-based learning, evidence-based medicine, and problem-based learning, imparting inquisitiveness among students to apply their knowledge and improve their skills. CBME includes many new ways of teaching such as early clinical exposure, the student-doctor method of clinical training, electives, skill development and training, and secondary hospital exposure, among other things. These are meant to help Indian medical graduates become good clinicians, leaders, communicators, lifelong learners, and professionals [3-5].

CBME includes learning in a stepwise manner from the attainment of knowledge to the demonstration of understanding of knowledge and performance of procedural skills under supervision to achieve the desired learning outcome, i.e., competency [6]. The Dreyfus Model explains an individual's progression for acquisition of skills is through a series of five levels: novice, advanced beginner, competent, proficient, and expert. As per the Dreyfus Model, a student needs to attain a level of competency through repeated practice over the years under supervision, followed by feedback for improvement. The learner acquires skills through trial and error, guided by the instructor [7].

Competency-based assessment (CBA) helps learners to achieve next-stage mastery rather than evaluation. It is longitudinal, often with low stakes, and involves direct observation, which helps to monitor the learner's progression, reduce examination anxiety, and assist in the generation of authentic valuable feedback to the learner. As an important part of CBAs, formative assessments (FAs) need to be taken often based on the needs of certain content, topics, or skills within the framework of competencies. Summative assessments supplement this process. The assessment process should be continuous, providing information for a continuous cycle of plan-do-study-act (PDSA) over time. Most commonly, CBA uses the Objective Structured Practical Examination (OSPE) or Objective Structured Clinical Examination (OSCE) to reduce examiner bias and enhance objectivity. It is a valid and reliable tool of assessment [8-10].

An FA with effective feedback about performance is crucial to improving the learner's outcome. "Feedback is personalized information based on direct observation, crafted and delivered so that receivers can use the information to achieve their best potential" [11]. Feedback given by the teacher should be specific on behavior, non-evaluative, and followed by confirmation of understanding and developing an action plan. Moreover, the student has to enhance their receptivity to the suggestions given by the teacher. Teachers expect students to implement feedback suggestions, thereby improving their learning process and outcome [11]. Lack of knowledge,, lack of sufficient professional competencies to deliver the feedback, and lack of time lead to improper delivery of feedback [12]. Weallans et al. proposed a composite model for providing feedback in clinical supervision [13]. The steps involved are: establishing and maintaining the educational alliance, observing performance, choosing the right time to initiate feedback sessions, asking for self-assessment, providing the right feedback, exploring the supervisor's view, and developing an action plan.

The involvement of teachers in FA, feedback, and active participation of students in the form of self-assessment and reflections makes FA and feedback impactful. However, a shortage of faculty, faculty resistance to change, poor resources, lack of motivation in students, lack of knowledge, and the importance of structured feedback make CBAs less effective and lead to a gap between students' performance and teachers' expectations [7-9]. This study aimed to determine how structured formative feedback training for faculty and the sensitization of students impact student performance during the FA of physiology practicals, as well as to gain insights from teachers and students regarding this.

## Materials and methods

This study was conducted at the Department of Physiology, Bharati Vidyapeeth (Deemed to be University) Medical College and Hospital, Sangli, Maharashtra, India. The study was approved by the Institutional Ethical Committee of Bharati Vidyapeeth (Deemed to be University) Medical College and Hospital (approval number: BV(DU)MC&H/Sangli/IEC/469/22, dated April 7, 2022). 

All the students of Phase I MBBS (2021-22 batch) and all seven faculty members from the Department of Physiology were selected as participants in this study. Written informed consent was obtained from those willing to participate after being explained the study design. All students with less than 75% attendance in the FA were excluded.

FAs were regularly conducted for hematology and the clinical practical in physiology as part of the CBME curriculum. These were done using OSPE/OSCE in small group teaching sessions with 6-10 students per teacher. By a blinding method, each student was asked to select any one Specific Learning Objective (SLO) from a given competency. Competencies evaluated during the FA of Physiology practicals include PY2.11, PY5.12, PY4.10, PY5.15, PY6.9, and PY10.11. A checklist was prepared as per SLO on competencies of hematology and clinical practical to be assessed, scoring 10 marks each (see Appendix A). This checklist was verified by the senior faculty of the Department of Physiology and the Medical Education Unit (MEU) of the Institute. It was shared with students a day prior to the assessment. During the FA, we asked students to perform according to the shared checklist. The teacher conducted a one-on-one assessment of the student's performance using a checklist and provided feedback.

**Figure 1:**
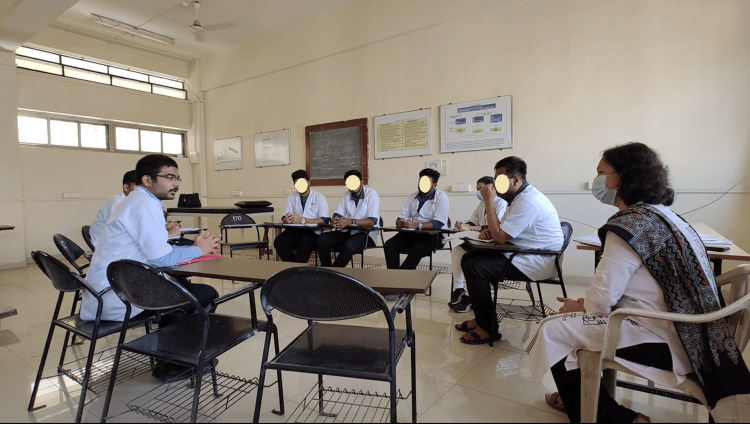
Focussed group discussion

The study was conducted in two parts between June 2022 and October 2022. The first part was conducted in the middle of the academic year after the students had finished their six FAs with feedback sessions on their performance. A focused group discussion (FGD) with video recording was conducted for faculty and students separately to determine their perception and insight of feedback given after the FA (Figure 1). It was conducted for 10 randomly selected students using a lottery method, equally distributed between high- and low-performance groups, who attended all six FAs. All seven faculty members from the Department of Physiology participated in the FGD. It was conducted in the presence of the MEU coordinator. The principal investigator acted as a moderator. Points for discussion were their knowledge, perception, awareness, and importance of structured formative feedback.

Following this, training and sensitization sessions were organized to teach faculty and students how to provide and receive structured feedback, respectively. It was conducted separately for faculty and students by well-experienced MEU faculty who had completed the Foundation for Advancement of International Medical Education and Research (FAIMER) fellowship. All faculty members in the Department of Physiology received training to provide structured formative feedback to students after FAs. This training included the usefulness and importance of FAs for learning, types of feedback, barriers to effective feedback, and delivery of effective structured feedback by using Pendleton’s model of delivering feedback. All the students were sensitized about the importance of FAs for learning, followed by structured feedback on their performance. We trained them on how to interpret, receive, internalize, and effectively use the feedback to enhance their performance.

**Figure 2:**
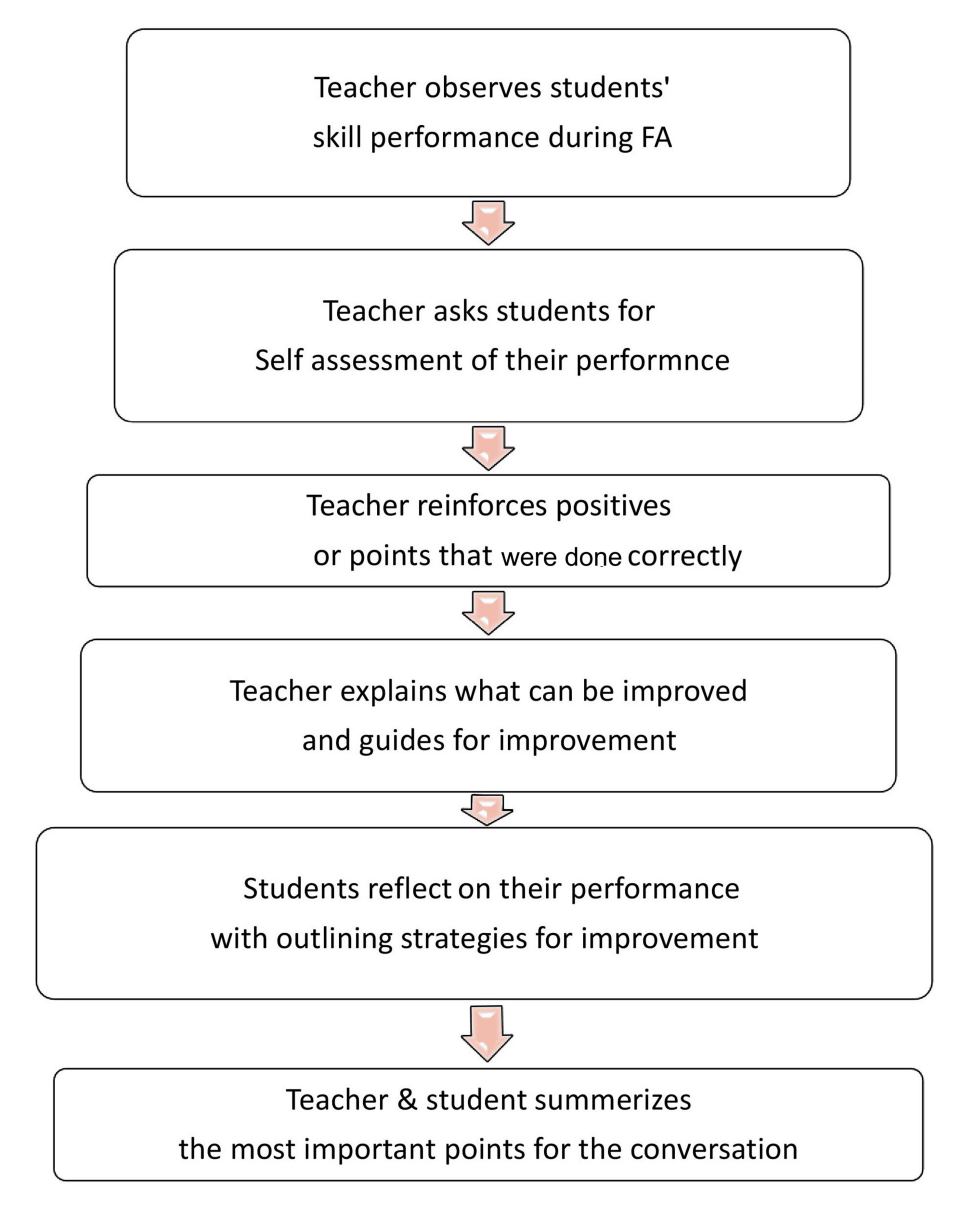
Steps used during delivery of structured formative feedback by using Pendleton’s Rules FA: formative assessment

After the training sessions of faculty and students, in the second part of the study, six FAs were conducted for hematology and clinical practicals. The trained faculty then used defined steps of Pendleton's method to provide structured one-to-one feedback to the students after the FA, following the steps given in Figure 2 [13,14].

A questionnaire was used to gather perceptions and attitudes regarding the delivery of structured formative feedback after the training session. Two separate questionnaires were prepared for faculty and students using existing literature with necessary modifications (see Appendix B, C). The questionnaires underwent validation and reliability tests. The reliability test revealed a Cronbach's Alpha value greater than 0.7, indicating good internal reliability. Faculty and students received these questionnaires as Google Forms (Google LLC, Mountain View, California, United States). A qualitative analysis was conducted and the perceptions were calculated as percentages. The OSPE/OSCE checklists (see Appendix D, E) were used to quantitatively analyze the students' FA scores before and after training sessions on structured formative feedback. We compared the scores before and after training sessions using paired t-tests.

## Results

FGDs with students revealed that they were aware of the importance of the feedback, but they were unable to internalize the mistakes and make necessary changes to improve their performance. They expressed their opinion that it was important to provide feedback in a suitable environment and in an appropriate way. In FGDs with teachers, it was observed that, despite being aware of the feedback process, many teachers did not follow the method of structured feedback. The reasons mentioned were giving feedback after every assessment and to each student is a time-consuming, stressful process. A total of 138 students responded to the questionnaire to assess perception and awareness about structured feedback delivered by teachers after every FA.

As shown in Table 1, 100% of students felt that immediate verbal feedback is a meaningful, appropriate, and effective tool, promotes active learning, and improves practical skills. About 95-98% of the students feel that structured feedback given in a stepwise manner is more effective and solution-oriented, and it was important to identify mistakes, help them improve their skills, and motivate them to learn the subject better. According to 53% of students, the whole feedback program was not stressful, while 23% of students stated that it was stressful. Students expressed mixed views on being pointed out only mistakes during feedback, with 46% disagreeing, 17% being neutral, and 37% agreeing. According to 69% of students, feedback sessions were not a waste of time. Surprisingly, 14% of students felt that receiving feedback was a waste of time. About 95% of students opined that the feedback was provided in a comfortable environment. About 75% of students agreed that it was delivered in a sandwich manner but 11% of students did not agree with it. They specifically mentioned that to reduce the gap between their performance and teachers' expectations, this feedback system should be used more often during revision practicals before the conduction of FA. Such a feedback system helped to build a good rapport with teachers. About 90% of students expressed that the time given for feedback was adequate and it was delivered in a friendly way, and it was up to their expectations, 93% of students expressed that the mention of weaknesses was non-threatening. According to about 88% of students, constructive feedback should be immediately given after FA.

**Table 1:** Perception and awareness of students towards structured formative feedback delivered after training session

Sr No	Questions	Responses, n (%)				
		Strongly agree	Agree	Neutral	Disagree	Strongly Disagree
Students' awareness regarding formative feedback						
1	Receiving feedback is important for making us understand our mistakes	86 (62.31%)	48 (34.78%)	04 (02.98%)	00 (00%)	00 (00%)
2	Feedback about my performance will help me to identify my shortcomings (practical skills)	82 (59.42%)	53 (38.40%)	03 (02.17%)	00 (00%)	00 (00%)
3	It will help to understand the gap between my performance and teachers’ expectations	76 (55.07%)	58 (42.02%)	04 (02.89%)	00 (00%)	00 (00%)
4	Feedback should be given after every formative assessment	66 (47.82%)	60 (43.47%)	12 (08.69%)	00 (00%)	00 (00%)
5	Feedback should be given immediately after the assessment	68 (49.27%)	55 (39.85%)	15 (10.86%)	00 (00%)	00 (00%)
6	Receiving feedback from teachers is waste of time	14 (10.14%)	06 (04.34%)	23 (16.66%)	26 (18.84%)	69 (50.00%)
Perception of students regarding the importance of feedback						
7	During feedback, my strengths and my weaknesses were mentioned in a non-threatening way	80 (57.97%)	49 (35.50%)	06 (04.34%)	01 (0.72%)	02 (01.44%)
8	Feedback will help me to reflect on my weakness	99 (71.73%)	38 (27.53%)	01 (0.72%)	00 (00%)	00 (00%)
9	Feedback helps to build a good rapport with the teacher	89 (64.49%)	42 (30.43%)	07 (05.07%)	00 (00%)	00 (00%)
10	The immediate verbal feedback program is a meaningful, appropriate, and effective learning tool for us	98 (71.01%)	40 (28.98%)	00 (00%)	00 (00%)	00 (00%)
11	The immediate verbal feedback program is interesting, will promote active learning, and improve practical skills.	99 (71.73%)	39 (28.26%)	00 (00%)	00 (00%)	00 (00%)
12	Feedback given at the time of revision tests is more effective than after the assessment results.	87 (63.04%)	43 (31.15%)	07 (05.07%)	01 (0.72%)	00 (00%)
Students' Reflection on the Process of Feedback						
13	Environment in which Feedback was provided was comfortable for me.	77 (55.79%)	55 (39.85%)	05 (03.62%)	00 (00%)	01 (0.72%)
14	Time given for feedback was adequate	64 (46.37%)	65 (47.10%)	07 (05.07%)	01 (0.72%)	01 (0.72%)
15	It was given in very friendly way.	76 (55.07%)	53 (38.40%)	06 (04.34%)	02 (01.44%)	01 (0.72%)
16	Feedback was given in sandwich manner i.e. positive + negative + positive	47 (34.05%)	58 (42.02%)	19 (13.76%)	09 (06.52%)	05 (03.62%)
17	Feedback given by teacher was only to point out my mistakes	25 (18.11%)	26 (18.84%)	23 (16.66%)	29 (21.01%)	35 (25.36%)
18	The whole feedback program was stressful.	18 (13.04%)	14 (10.14%)	33 (23.91%)	34 (24.63%)	39 (28.26%)
19	The whole feedback program has met my expectation	49 (35.50%)	71 (51.44%)	16 (11.59%)	00 (00%)	02 (01.44%)
20	Structured feedback is more effective; solution oriented and will help to improve my skills.	76 (55.07%)	57 (41.30%)	04 (02.89%)	01 (0.72%)	00 (00%)
21	Feedback motivates us to learn the subject better	80 (57.97%)	55 (39.85%)	03 (02.17%)	00 (00%)	00 (00%)

A total of seven faculty from the Department of Physiology were actively involved in conducting FAs and delivering structured formative feedback. All participating faculty liked the idea of giving structured feedback after FA and agreed/strongly agreed that feedback should be used for both positive and negative performance points in a non-threatening manner (Table 2). At the same time, they felt that one-to-one feedback after every FA was time-consuming and needed commitment. According to 85% (n=6) of faculty, the process of giving feedback was easy and it helped to identify the unclear points and gaps in the understanding of students, which in turn assisted self-evaluation of teaching. The sensitization training and FGD sessions motivated and guided them to provide feedback in a structured manner. These structured feedback sessions helped minimize mistakes by students and improved their confidence as well as their performance in the demonstration of skills, as noticed in subsequent summative examinations.

**Table 2:** Perception and awareness of Faculty regarding structured formative feedback after training session

Sr. No.	Questions	Response, n (%)				
		Strongly agree	Agree	Neutral	Disagree	Strongly disagree
1	Giving feedback after formative assessment is a good idea	3 (42.85%)	4 (57.14%)	0 (0%)	0 (0%)	0 (0%)
2	Feedback should be provided only for positive points of performance	0 (0%)	0 (0%)	2 (28.57%)	3 (42.85%)	2 (28.57%)
3	Feedback should be provided for both positive and negative points of performance	4 (57.14%)	3 (42.85%)	0 (0%)	0 (0%)	0 (0%)
4	Feedback should be provided in a non-threatening way	6 (85.71%)	1 (14.28%)	0 (0%)	0 (0%)	0 (0%)
5	This process of giving feedback is easy to carry out	2 (28.57%)	4 (57.14%)	0 (0%)	1 (14.28%)	0 (0%)
6	This approach enhances the valuable exchange of ideas between teacher and student	2 (28.57%)	4 (57.14%)	1 (14.28%)	0 (0%)	0 (0%)
7	This exercise has increased my knowledge of how to give feedback to students	3 (42.85%)	4 (57.14%)	0 (0%)	0 (0%)	0 (0%)
8	Verbal one-to-one feedback is time-consuming	1 (14.28%)	5 (71.42%)	0 (0%)	1 (14.28%)	0 (0%)
9	Feedback helped me in self-assessment	3 (42.85%)	3 (42.85%)	1 (14.28%)	0 (0%)	0 (0%)
10	I am now motivated to give feedback to students	4 (57.14%)	2 (28.57%)	1 (14.28%)	0 (0%)	0 (0%)

We evaluated the impact of the structured feedback training session on students' performance during hematology and clinical practicals. This evaluation was done by comparing the scores of FAs for both giving and receiving structured formative feedback, before and after the training session. As shown in Table no. 3, a p-value < 0.001 indicates a significant improvement in scores in FAs conducted after a training session.

**Table 3:** Comparative analysis of scores of students obtained during FA conducted before and after training session *paired t-test FA: formative assessment

Scores of students during FA	Mean + SD	t-value	p-value*
Before training session	31.93 + 3.66	29.46	< 0.001
After training session	43.1 + 3.38		

## Discussion

FA using OSPE or OSCE is a more consistent way to evaluate students, as it is more objective, reduces examiner bias, and helps generate valuable feedback [10]. Therefore, in the current study, we conducted FAs using OSPE or OSCE followed by feedback to the students on their performance in Physiology practicals.

In our study, during FGD, it was observed that there was a gap between students and teachers regarding receiving feedback and delivering input, respectively. Training sessions for teachers helped them deliver structured feedback in a stepwise manner, in the proper surroundings, and at the proper time. Training sessions for students sensitized them about the importance of feedback from teachers on their performance and making necessary changes in performance, as there was an improvement in scores in FAs conducted after training sessions than before. 

In a study by Rao Gutti et al., they looked at how FA and feedback affected the summative assessment of a hypertension clinico-social case in Phase 3 part I MBBS students working as community medicine residents [15]. As in the current study, they found a significant difference in total scores, mean scores, and scoring percentage of students before and after the intervention. The majority of faculty and students opined that FA and feedback were very useful in learning. After this session, some students were playful while others found this stressful and time-consuming. Also, some found differences in the conduct of FA and feedback by faculty [15].

Mngomezulu et al., in their study on Grade 10 Physical Sciences teachers, recommended necessary training for teachers to effectively utilize formative feedback and improve learners’ academic achievement in physical sciences, which helps in their professional development [12]. According to them, formative feedback in teaching and learning physical sciences should be precise, well-timed, and supportive of students' learning. Overcrowding, lack of resources and material, and the inability of students to utilize formative feedback to improve their learning are some issues that can be minimized by training teachers.

Well-timed, private, and verbal feedback is effective and promotes the development of action plans by learners [16]. The delivery of structured formative feedback using the Pendleton method benefits learners as they evaluate their performance and identify areas of improvement. Self-assessment by learners develops insight to improve performance and makes teachers comfortable to deliver negative feedback. Regular formative feedback fosters a growth mindset in students, enabling them to view challenges and failures as opportunities for improvement and learning. Students’ ability to self-assess and self-regulate led to improvements in personal and professional development, self-regulation, and a developed leadership mindset [14,17]. Teachers get training on how to use this method to give formative feedback, which helps students do better on FAs in physiology practicals by improving their skill performance.

Mentoring, feedback, and self-reflection are important in the professional development of Indian medical graduates. Feedback helps in the professional development of the trainee by directly observing the academic performance [18]. Multiple encounters or checkpoints for FA and tailoring the individual feedback according to the performance help to identify students' learning difficulties and gaps. Effective feedback reinforces good practice, promotes self-reflection, and builds up qualities like self-directed learning, lifelong learning, and good communication to be a good clinician; however, improper delivery of feedback can be hazardous, resulting in a negative impact and demoralizing students' performance. However, feedback is the less noticeable part of the assessment and needs standardization [19].

Good educators should provide education that modifies student thinking and behavior positively and fosters generalized learning improvement; excellent formative feedback enables this. Despite the challenges of designing and delivering robust feedback with a growing student population and limited time, teachers should consistently provide descriptive, purposeful feedback in a timely manner. The barriers to the delivery of high-quality feedback are ineffective methods of feedback delivery, hesitance to give negative feedback, inability to identify specific issues, and delay in completing evaluations and feedback [16,19,20].

Therefore, training and faculty development programs help overcome the barriers to effective feedback by motivating teachers to deliver structured feedback regularly. The study's effects were visible in the students' improved performance on the summative exam conducted after four to five months. External assessors expressed satisfaction with the students' performance and efforts. The authors intend to conduct a questionnaire-based survey with the same participants in the future to explore the long-term effects and determine whether the structured feedback has enhanced their interactions with patients during clinical assignments.

Intentional and consistent formative feedback by faculty enhances students' learning potential, self-assessment, and self-regulation, it identifies and corrects learning gaps, reduces anxiety, and builds up confidence, improving their skill performance in both formative and summative assessments [17,19,20].

The study has several limitations, including a small sample size, potential self-reporting bias in the questionnaire, a limited number of faculty participants in the subject area, a short study duration, and the focus on only one college. Future research could explore the long-term effects of structured formative feedback delivered after training on summative assessments. Multicentric studies should be conducted to achieve more accurate results.

## Conclusions

There was a significant improvement in scores in FAs in Physiology practicals after the delivery of structured formative feedback, following training to faculty and sensitization of students. Thus, feedback given in a structured manner helps learners to assess and monitor their learning, develop insight, and provide ways of improving. This approach fosters self-directed learning, lifelong learning, and communication skills apart from psychomotor skills, essential to becoming effective clinicians.

Our study findings indicate that the structured feedback training program for faculty can lead to positive changes in their behavior, knowledge, and attitude. This program helps to overcome barriers and motivates faculty to incorporate feedback practices regularly as a part of the assessment process. Barring the constraints of time, one-to-one assessment and structured feedback appear to be effective tools in the teaching and learning process. Careful planning and the use of OSPE/ OSCE stations with checklists for FA can help manage time constraints and lack of resources.

## Figures and Tables

**Figure 3:**
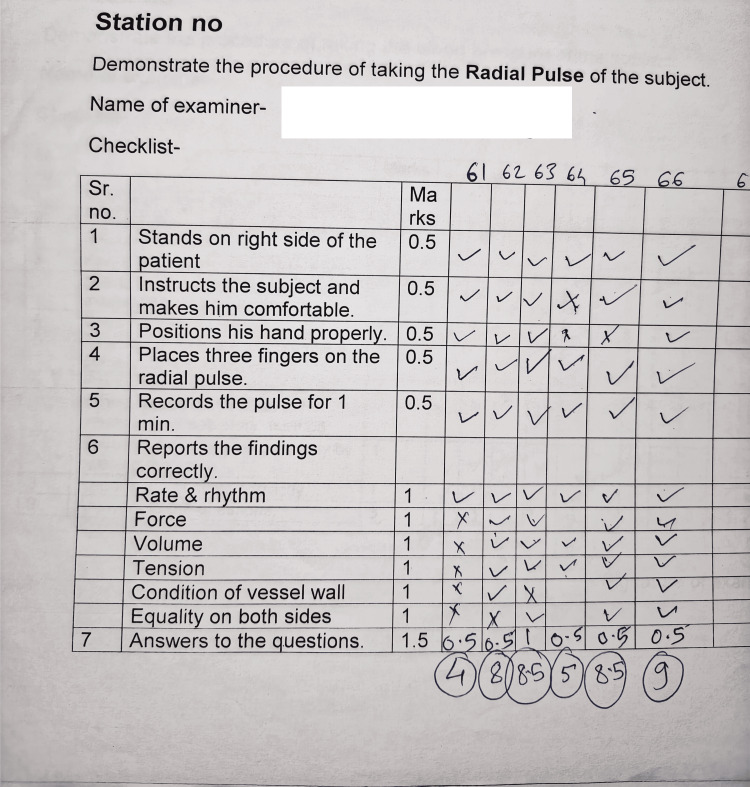
OSCE checklist used for formative assessment (Proof of OSCE) OSCE: objective structured clinical examination

**Figure 4:**
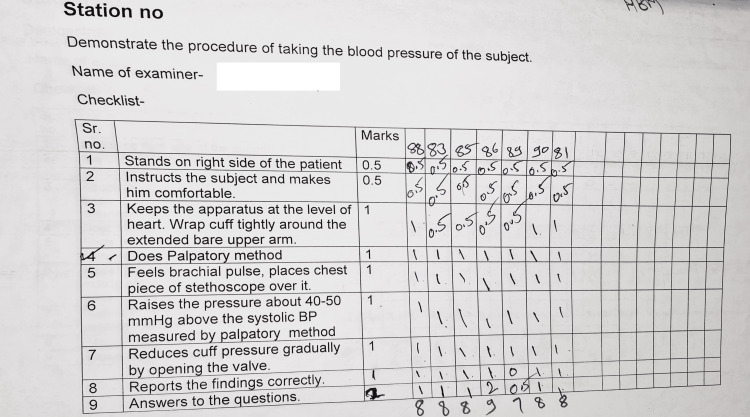
OSCE checklist used during formative assessment (proof of OSCE conducted) OSCE: objective structured clinical examination

## Appendices

Appendix A

**Table 4:** Checklist for OSCE for Competency PY 4.10 OSCE: objective structured clinical examination

Topic: Gastro-intestinal Physiology PY4.10: Demonstrate the correct clinical examination of the abdomen in a normal volunteer or simulated environment		
Sr. no	STATION 1: Demonstrate palpation of spleen	marks
1	Stands on the right side of the patient	0.5
2	Instructs the subject.	0.5
3	Makes the patient comfortable.	0.5
4	Exposes the abdomen of the patient.	0.5
5	Warms hands.	0.5
6	Asks to fold the knees.	1
7	Asks the subject to breathe in deeply.	0.5
8	At the height of inspiration presses the right hand on the patient's abdomen.	0.5
9	Starts palpation from the right iliac fossa.	0.5
10	Border of the palpating hand should be parallel to the border of the palpating organ.	1
11	Reaches left hypochondrium.	0.5
12	Movements of the hand are smooth and not poking.	0.5
13	Reports correctly	1
14	Answers to the questions	2
Total		10
Sr. no	STATION 2: Demonstrate palpation of liver	Marks
1	Stands on the right side of the patient	0.5
2	Instructs the subject.	0.5
3	Makes the patient comfortable.	0.5
4	Exposes the abdomen of the subject.	0.5
5	Warms hands.	0.5
6	Asks to fold the knees.	1
7	Asks the subject to breathe in deeply.	0.5
8	At the height of inspiration presses the right hand on abdomen.	0.5
9	Starts palpation from the right iliac fossa.	0.5
10	Border of the palpating hand should be parallel to the border of the palpating organ.	1
11	Reaches right hypochondrium.	0.5
12	Movements of the hand are smooth and not poking	0.5
13	Reports correctly	1
14	Answers to the questions	2
Total		10
Sr. no	STATION 3: Demonstrate palpation of kidneys.	Marks
1	Stands on the right side of the patient	0.5
2	Instructs the subject.	0.5
3	Makes the patient comfortable.	0.5
4	Exposes the abdomen of the subject.	0.5
5	Warms hands.	0.5
6	Asks to fold the knees.	1
7	Asks the subject to breathe in deeply.	0.5
8	Places left hand posteriorly in the loin	1
9	Places right hand in the lumbar region anteriorly	1
10	At the height of inspiration presses right hand upwards and inwards and left hand upwards.	1
11	Movements of the hand are smooth and not poking	0.5
12	Reports correctly	1
13	Answers to the questions	1.5
Total		10
Sr. no	STATION 4: Demonstrate the procedure of eliciting horseshoe-shaped dullness in the subject.	Marks
1	Stands on the right side of the patient	0.5
2	Instructs the subject	0.5
3	Makes the patient comfortable.	0.5
4	Exposes the abdomen of the subject.	0.5
5	Warms hands.	0.5
6	Asks to fold the knees.	1
7	Places both hands as in percussion on the abdomen in the center	1
8	Start percussing from the umbilicus towards the periphery.	1
9	Does percussion in all directions to mark horseshoe-shaped dullness at the periphery	1
10	Movements of the hand are smooth and not poking	0.5
11	Reports correctly	1
12	Answers to the questions	2
Total		10
Sr. no	STATION 5: Demonstrate the procedure of eliciting shifting dullness in the subject	Marks
1	Stands on the right side of the patient.	0.5
2	Instructs the subject	0.5
3	Makes the patient comfortable.	0.5
4	Exposes the abdomen of the subject.	0.5
5	Warms hands.	0.5
6	Asks to fold the knees.	1
7	Places both hands as in percussion on the abdomen in the center	1
8	Start percussing and then moves laterally till the dull note appears.	0.5
9	Asks the subject to roll over such that the lateral part of the abdomen now lies on top.	0.5
10	Percusses again, ensuring that the hand does not move away from the area.	1
11	Movements of the hand are smooth and not poking	0.5
12	Reports correctly	1
13	Answers to the questions	2
Total		10
Sr. no	STATION 6: Demonstrate the procedure of eliciting a fluid thrill in the subject.	Marks
1	Stands on the right side of the patient	0.5
2	Instructs the subject	0.5
3	Makes the patient comfortable	0.5
4	Exposes the abdomen of the subject	0.5
5	Warms hands	0.5
6	Asks to fold the knees	1
7	Places one hand flatly on the side of the abdomen in the right position	1
8	Asks the subject to put the ulnar side of their hand in the midline	1
9	Taps the other side of the abdomen	1
10	Movements of the hand are smooth and not poking	0.5
11	Reports correctly	1
12	Answers to the questions	2
Total		10

Appendix B

**Table 5:** Questionnaire to assess perception of students about structured formative feedback delivered after training session

	Question	Strongly agree	Agree	Neutral	Disagree	Strongly Disagree
	Perception of students regarding formative feedback					
1	Receiving feedback is important for making us understand our mistakes					
2	Feedback about my performance will help me to identify my shortcomings (practical skills).					
3	It will help to understand gap between my performance and teachers expectations					
4	Feedback should be given after every formative assessments					
5	Feedback should be given immediately after assessment					
6	Receiving feedback from teacher is waste of time					
	Importance of feedback					
7	During feedback my strengths were mentioned and my weaknesses were mentioned in nonthreatening way					
8	Feedback will help me to reflect on my weakness					
9	Feedback helps to build good rapport with the teacher					
10	The immediate verbal feedback program is meaningful, appropriate and effective learning tool for us					
11	The immediate verbal feedback program is interesting, will promotes active learning and improves practical skills.					
12	Feedback given at the time of revision tests is more effective than after the assessment results.					
	Reflection on feedback					
13	Environment in which Feedback was provided was comfortable for me.					
14	Time given for feedback was adequate					
15	It was given in very friendly way.					
16	Feedback was given in sandwich manner i.e. positive + negative + positive					
17	Feedback given by teacher was only to point out my mistakes					
18	The whole feedback program was stressful.					
19	The whole feedback program has met my expectation					
20	Structured feedback is more effective; solution oriented and will help to improve my skills.					
21	Feedback motivates us to learn the subject better					

Appendix C

**Table 6:** Questionnaire to assess perception of faculty regarding structured formative feedback delivered after training session

Sr. No.		Strongly agree	Agree	Neutral	Disagree	Strongly Disagree
1	Giving feedback after formative assessment is a good idea					
2	Feedback should be provided only for positive points of performance					
3	Feedback should be provided for both positive and negative points of performance					
4	Feedback should be provided in non threatening way					
5	This process of giving feedback is easy to carry out					
6	This approach enhances valuable exchange of ideas between teacher and student					
7	This exercise has increased my knowledge on how to give feedback to students					
8	Verbal one to one feedback is time consuming					
9	Feedback helped me in self assessment					
10	I am now motivated to give feedback to students					

Appendix  D

Appendix E
